# The Ec-NhaA antiporter switches from antagonistic to synergistic antiport upon a single point mutation

**DOI:** 10.1038/srep23339

**Published:** 2016-03-29

**Authors:** Manish Dwivedi, Shahar Sukenik, Assaf Friedler, Etana Padan

**Affiliations:** 1 Department of Biological Chemistry, Alexander Silberman Institute of Life Sciences, Givat Ram, Jerusalem-91904, Israel; 2 Institute of Chemistry, the Hebrew University of Jerusalem, Edmond J. Safra Campus, Givat Ram, Jerusalem-91904, Israel

## Abstract

The Na^+^, Li^+^/H^+^ antiporter of *Escherichia coli* (Ec-NhaA) maintains pH, Na^+^ homeostasis in enterobacteria. We used isothermal titration calorimetry to perform a detailed thermodynamic analysis of Li^+^ binding to Ec-NhaA and several of its mutants. We found that, in line with the canonical alternative access mechanistic model of secondary transporters, Li^+^/H^+^ binding to the antiporter is antagonistically coupled. Binding of Li^+^ displaces 2 H^+^ from the binding site. The process is enthalpically driven, the enthalpic gain just compensating for an entropic loss and the buffer-associated enthalpic changes dominate the overall free-energy change. Li^+^ binding, H^+^ release and antiporter activity were all affected to the same extent by mutations in the Li^+^ binding site (D163E, D163N, D164N, D164E), while D133C changed the H^+^/Li^+^ stoichiometry to 4. Most striking, however, was the mutation, A167P, which converted the Ec-NhaA antagonistic binding into synergistic binding which is only known to occur in Cl^−^/H^+^ antiporter.

Living cells are critically dependent on processes that regulate intracellular pH, Na^+^ and volume. Na^+^/H^+^ antiporters, which play a primary role in these homeostatic mechanisms^1^,^2^, are found in the cytoplasmic and intracellular membranes of most organisms. Certain Na^+^/H^+^ antiporters have long been drug targets^3^ because they are involved in cardiac failures and other disorders (reviews in^4^,^5^,^6^).

Ec-NhaA, the principal Na^+^, Li^+^/H^+^ antiporter in *Escherichia coli*, is responsible for intracellular Na^+^ and H^+^ homeostasis^7^. Ec-NhaA homologues have recently been implicated in the virulence of pathogenic bacteria^8^,^9^ and in human essential hypertension^10^ as well as diabetes^11^. Ec-NhaA is characterized by exceptionally high transport activity^12^, a stoichiometry of 2H^+^/Na^+ ^13 and strong pH dependence^12^, a property shared with other prokaryotic^7^ and eukaryotic Na^+^/H^+^ antiporters (reviewed in^14^,^15^,^16^,^17^).

The crystal structure of down-regulated Ec-NhaA at acidic pH^18^ (Fig. 1a) shows that the protein is made up of 12 trans-membrane helices (TMs). Six of these TMs form a highly-conserved core domain, composed of two structurally-related helix bundles (TMs III, IV, V and TMs X, XI, XII) that are topologically inverted with respect to each other^18^ (Fig. 1a,b). TMs IV and XI are each interrupted by an unwound chain that crosses the other chain in the middle of the membrane, leaving two short helices oriented either towards the cytoplasm (c) or towards the periplasm (p) (IVc, IVp, and XIc, XIp, respectively and Fig. 1 18). This non-canonical TM assembly is termed the NhaA fold. This unique fold creates a delicately-balanced electrostatic environment in the middle of the membrane at the ion-binding site, and this environment is likely to play a critical role in cation exchange activity^18^. The number of secondary transporters known to share the NhaA fold is steadily increasing^19^,^20^,^21^,^22^,^23^,^24^.

Solving the Ec-NhaA structure has enabled mutational and biochemical/biophysical data to be interpreted in a rational way^25^. Two functional regions have been identified: (a) a cluster of amino acyl side chains that are involved in pH regulation, localized outside the Na^+^/Li^+^ binding site^2^,^26^,^27^; and (b) a catalytic region containing the Na^+^/Li^+^ binding site, located in the core domain (Fig. 1b). Evolutionary conservation analysis^28^, location in the crystal structure^18^, and mutagenesis results have shown that Asp163 and Asp164 are essential constituents of the antiporter’s Li^+^/Na^+^ binding site (Fig. 1). Specifically, the Cys replacement mutations D163C and D164C abolish growth in high Na^+^/Li^+^ selective media, in addition to Na^+^/Li^+^ transport activity^29^,^30^ (Table S1) and Li^+^ binding^31^.

Electrophysiology studies^32^ have revealed that the mechanism of Na^+^, Li^+^/H^+^ antiport is compatible with the canonical alternate accessibility mechanism of secondary transporters^33^. In its simplest form, the alternate accessibility model describes a shift between two conformations, one in which the ligand is accessible to the binding site from the cytoplasm, and another in which the ligand is accessible from the periplasm. In the Ec-NhaA antiport cycle, the ligands share a single binding site so that they are expected to bind antagonistically and cannot bind at the same time to the antiporter. We have previously identified the Li^+^ binding site of Ec-NhaA (Asp163, Asp164, Asp133) using Isothermal Titration Calorimetry (ITC). A similar binding site was identified for Na^+^ indirectly (by mutagenesis) because it has ten folds lower affinity to Ec-NhaA than Li^+^ and did not give a signal in ITC^31^.

Although the studies cited above have provided important advances in the understanding of the antiport mechanism of NhaA, very little is known about the thermodynamic details of the process and its energy-coupling mechanism. Several important open questions include the following: How does the electrochemical potential of an ion moving down its gradient across the membrane drive the up-hill active transport of the second substrate? Where does H^+^ pass through the membrane, and where is the H^+^ binding site?

ITC has recently been used to trace H^+^ in the reaction cycle of Ec-CLC, the anionic Cl^−^/H^+^ antiporter of *E. coli*^34^. These studies revealed that the Ec-CLC mechanism differs from that of the canonical secondary transporters; it exhibits synergistic rather than antagonistic ligand binding. Herein, we followed this approach to investigate the coupling mechanism of Li^+^ and H^+^ in the Ec-NhaA antiporter. We found that, in wild-type (WT) Ec-NhaA, binding of Li^+^ is linked to release of H^+^ from Ec-NhaA and the binding is antagonistic, as required by the canonical alternative accessibility mechanism of secondary transporters. In particular, binding of Li^+^ displaced 2 H^+^ from the binding site. We also used mutations in the Li^+^ binding site (D163E, D163N, D164N, D164E) and observed that Li^+^ binding, H^+^ release and antiporter activity were all affected to the same extent. Moreover, the mutation D133C changed the H^+^ /Li^+^ stoichiometry to four. Surprisingly, the mutation A167P changed the antagonistic binding of H^+^/Li^+^ to a synergistic binding of 6 H^+^ to the antiporter, thereby eliminating the vast mechanistic difference between Ec-NhaA and the anionic antiporter, Ec1-CLC.To further corroborate the mechanistic changes induced by these mutations, we evaluated changes in the absorbance spectrum of a pH indicator that were attributable to antiporter action. The results of these experiments fully supported the results we obtained with thermodynamic analysis.

## Results

We used ITC to determine how Li^+^ binding to WT Ec-NhaA or mutants is linked to the exchange of protons. As described previously^34^, the total enthalpy measured during an ITC experiment is given by the following equation:


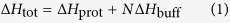


Where Δ*H*_tot_ is the total enthalpy measured during an ITC experiment, which is the sum of the enthalpies arrising from protein-specific reactions denoted Δ*H*_prot_ (Li^+^ binding, conformational changes and protonation or deprotonation of the protein) and the ionization enthalpy of the buffer, Δ*H*_buff_ (resulting from the buffer donating or accepting the protons exchanged by the protein^35^,^36^,^37^). *N* is the number of protons exchanged between the protein and buffer per Li^+^ binding event. Δ*H*_buff_ is a buffer-specific quantity and can be directly measured in the absence of protein (Fig. S1). Therefore, the slope of the linear relationship between Δ*H*_tot_ and Δ*H*_buff_ is the stoichiometry of coupled Li^+^ and H^+^ binding. If substrate binding to Ec-NhaA is not coupled, then Δ*H*_tot_ should not vary when measured in buffers with different Δ*H*_buff_ values, so that *N* should be 0. Conversely, a direct coupling with substrate binding would result in a variable Δ*H*_tot_ value. A negative slope indicates that Li^+^ binding induces release of a proton from the antiporter (antagonistic binding), whereas a positive slope indicates that Li^+^ binding promotes protonation of the protein (synergistic binding). The intercept of the line, Δ*H*_prot_, is the enthalpy change associated with protein binding of Li^+^, release of H^+^, and any conformational re-arrangement that may occur as a result of these processes. Δ*G*_tot_ and *T*Δ*S*_tot_ and the respective values for the buffers were calculated, respectively, by using the following formulas:


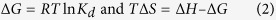


*T*Δ*S*_prot_ and Δ*G*_prot_ were calculated by subtracting the buffer’s value from the total value.

### Li^+^ Binding to WT Ec-NhaA Induces Deprotonation

Binding of Li^+^ to WT Ec-NhaA was measured in four buffers: BTP, glycyl-glycine, TAPS and Tris. The pKa values of these buffers lie in the pH-range of 8.0 to 9.0, the required pH range for activation of Ec-NhaA^12^ (Fig. S1b). The ionic strength of each reaction mixture was 165 mM. The liberated heats were fit to single-site-binding isotherms^38^. The Δ*H*_buff_ values of these buffers range from 10 to 12.5 kcal mol^−1^ (Fig. S1). Importantly, Li^+^ binds to WT Ec-NhaA with comparable affinities in all four buffers (Table 1), indicating that the buffer molecules do not interact with the Li^+^ binding sites. We have previously obtained similar results regarding Li^+^ binding to WT Ec-NhaA in BTP^31^.

The results show that Δ*H*_tot_ for WT Ec-NhaA decreases monotonically with increasing change in the ionization enthalpy of the buffers (Fig. 2 and Table 1): the Δ*H*_tot_ in BTP (−11.8 ± 1.2 kcal mol^−1^) was smaller than that in glycyl-glycine (−12.8 ± 0.6 kcal mol^−1^), which was smaller than in TAPS (−13.5 ± 0.4 kcal mol^−1^), and the largest Δ*H*_tot_ was observed in Tris (−16.5 ± 0.6kcal mol^−1^). Hence, Li^+^ binding to the protein induces deprotonation of Ec-NhaA, indicating that substrate binding is linked and antagonistic; at equilibrium, either H^+^ or Li^+^ binds to the protein, but the two ions do not bind simultaneously.

### Two Protons Leave WT Ec-NhaA for Every One Li^+^-Binding Event

The binding stoichiometry of the ligands was found to be *N* = −2.1 ± 0.1 (Fig. 2 and Table 1). Thus, for roughly every Li^+^-binding event, two protons are released from Ec-NhaA. The 2H^+^/1Li^+^ binding stoichiometry found here by ITC for Ec-NhaA is identical to the antiporter’s transport stoichiometry of 2H^+^/1Na^+^, previously determined biochemically^13^ (see Discussion). This identity strongly suggests that the linked events of Li^+^ binding and H^+^ release are part of the Ec-NhaA exchange cycle. However, an alternative explanation could be that the released heat reflects deprotonation of one or more residues outside the cation pathway, such that the identical stoichiometries are merely a coincidence. Thus, to determine whether this linkage is a property related to the protein function, we used mutations that alter the transport cycle of Ec-NhaA at different steps and investigated how they affect the stoichiometry of binding.

### Mutations in the Li^+^ Binding-Site Identically Decrease Li^+^ Binding and H^+^ Release

Cys-replacement mutations in the active site have previously been shown to abolish both growth in high Na^+^/Li^+^ selective media and Na^+^/Li^+^ transport activity in everted membrane vesicles^39^ (Table S1). Here, we observed that mutations D163E, D163N and D164N behaved like the Cys replacements (Table S1 and Fig. 2). However, D164E grew under high Na^+^ stress conditions at neutral pH and showed significant antiport activity, although with higher apparent *K*m for both Na^+^ and Li^+^ as compared to the WT Ec-NhaA^39^ (Table S1). Moreover, the mutations that were lethal in selective growth media also eliminated Li^+^ binding to Ec-NhaA in all buffers used. However, mutant D164E showed Li^+^-coupled H^+^ release with a 2H^+^/Li^+^ stoichiometry similar to that of the WT but with a lower affinity (4–6-fold higher *K*d; Table 1). Taken together, our results imply that each of the investigated mutations in Asp163 and Asp164 affects the ability of the protein to bind Li^+^ and release H^+^ to an identical extent.

Note that most of the mutations used in this study are Cys replacements rather than Ala replacements. Cys replacements are routinely used in biochemical and biophysical studies of membrane proteins, because they can undergo site-directed chemical modification by various sulfhydryl-reagents^40^. Such chemical modification is used to test effects on functionality, to estimate accessibility of the replaced residue across the membrane, or to determine distances between site-directed Cys-replacements.

### The D133C Mutation Changes the H^+^/Li^+^ Stoichiometry of Ec-NhaA

**Figure 1:**
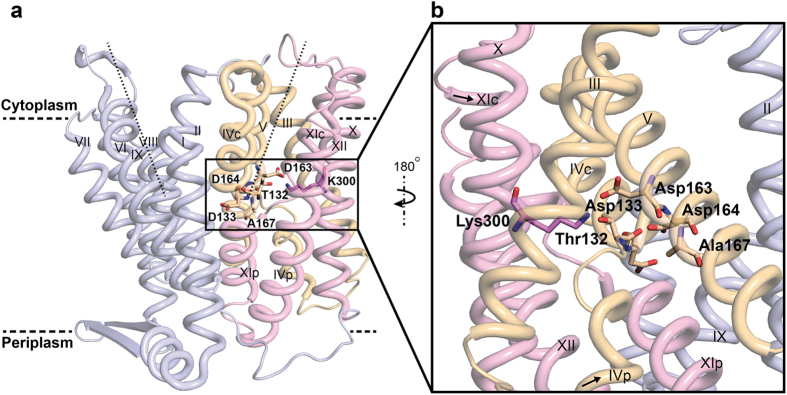
The crystal structure of Ec-NhaA and amino acid residues in or around the Li^+^ binding site. (**a**) The Ec-NhaA crystal structure^21^ is viewed parallel to the membrane (broken line) as a tube representation. The functionally-important amino acid residues are indicated in sticks, the TMs with Roman numerals, and the inward-facing funnel with a thin black line. The topologically-inverted repeats in the core, TMs III, IV, V and X, XI XII, are colored wheat and light pink respectively. (**b**) A blow-up showing the unique structural fold of Ec-NhaA in which TMs VI and XI of the inverted repeat (straight arrows mark the topologically-inverted N-termini) are interrupted by extended chains that cross each other in the middle of the membrane.

Thr132, Asp133 and Lys300 are also part of the substrate-binding site of Ec-NhaA^31^ (Fig. 1). We could not use ITC to measure Li^+^ binding to mutant T132C or mutants in K300, because in DDM micelles, the purified T132C protein was unstable and the mutants of K300 aggregated^31^. Our analysis for D133C indicated that the mutant exhibits Na^+^, Li^+^/H^+^ antiporter activity, although with a high apparent *K*m for Na^+^/Li^+^ (Table S1). It is stable in detergent micelles and binds Li^+^ as has previously been shown^31^, with the thermodynamic parameters summarized in Fig. 3 and Table 1. In all buffers, the *K*d of D133C for Li^+^ was about 7 times higher than that of the WT, and the H^+^/Li^+^ stoichiometry was 4, in contrast to 2 for the WT.

To test whether the changes induced by mutation D133C represent increased coupling of H^+^ via the Ec-NhaA-D133C protein, we introduced into D133C the mutation D164N, which inactivates the active-site^39^. The resulting double mutantEc-NhaA-D133C-D164N did not grow on high Na^+^/Li^+^ selective media and did not show any ITC signal upon addition of Li^+^ to the ITC reaction mixture, similarly to the mutant D164N (Fig. 3e). These results imply that the activity of the Ec-NhaA-D133C protein is responsible for the antiporter’s increased H^+^/Li^+^ stoichiometry (as compared to that of the WT).

### The A167P Mutation Changes the Antagonistic Binding of WT Ec-NhaA into Synergistic Li^+^/H^+^ Binding

**Figure 2:**
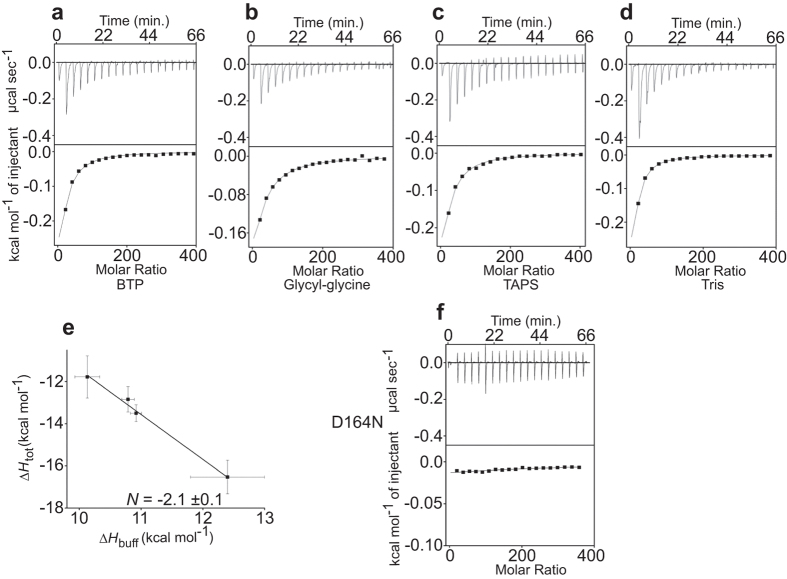
Antagonistic binding of H^+^ and Li^+^ to WT Ec-NhaA. (**a–d**) Top panels: Heat liberated when a 40-mM LiCl solution is injected into the experimental chamber containing WT Ec-NhaA (21–23 μM) and (**a**) BTP buffer, (**b**) glycyl-glycine, (**c**) TAPS, (**d**) Tris. Each downward deflection corresponds to one injection. Bottom panels: The area underneath each deflection is integrated and represents the total heat exchanged (squares) Bottom panels. Black lines are the best fits to a single-site binding isotherm. Averaged thermodynamic parameters are reported in Table 1. (**e**) Plot of the total enthalpy, Δ*H*_tot_, of Li^+^ binding as a function of the enthalpy of buffer ionization, Δ*H*_buff_ (Fig. S1). Squares, experimental data; solid line, fit to equation (1) with Δ*H*_prot_ = −10 ± 2 kcal/mol and N = 2.1 ± 0.1. (**f**) An example of an experiment done with the mutant Ec-NhaA-D164N in BTP buffer. The errors for the experimental points are the s.e.m. from several repeats, and those on Δ*H*_prot_ (Table 1 (b)) and N represent the uncertainty of fit.

The A167P mutation is located in TM V, outside but near the active site (Fig. 1 41). A167P shows substantial Na^+^, Li^+^/H^+^ antiporter activity in isolated membrane vesicles both at neutral pH and at alkaline pH, with an apparent *K*m similar to that of the WT (Table S1). The *K*d for Li^+^ binding was similar to that of the WT, but surprisingly, instead of showing the antagonistic Li^+^/H^+^ binding of the WT (Fig. 2), the A167P antiporter exhibited synergistic binding of Li^+^ and 6H^+^; Δ*H*_tot_ increased monotonically with the ionization enthalpy of the buffers, and at equilibrium H^+^ and Li^+^ were bound simultaneously to the protein (Fig. 4 and Table 1).

To test whether the absorbed heat by mutant A167P reflects protonation of residues related to the H^+^ pathway of the antiporter or protonation of one or more residues outside the cation pathway in a mere coincidence, we constructed the double mutant Ec-NhaA-A167P-D164N. Mutants D164N^39^ and A167P-D164N (Table S1) are lethal in high-Na^+^/Li^+^ selective media (Table S1). The mutant protein A167P-D164N did not show any ITC signal upon addition of Li^+^ to the reaction mixture (Fig. 4e), implying that indeed the mutant A167P synergistically binds Li^+^ and H^+^.

### Direct Determination of Li^+^-Induced H^+^-Release or Uptake by the Antiporter Reproduced the Thermodynamic (ITC) Results

To independently assess the conclusions derived from the thermodynamic analysis, we directly measured the pH of the medium following the Li^+^ injection. A purified Ec-NhaA variant, prepared as in the ITC experiment, was washed twice and resuspended in a reaction medium devoid of buffer but containing a pH indicator. For each protein, we used a pH indicator with pH sensitivity that corresponded well to the H^+^ movement in the medium; specifically, Cresol Red was used for WT Ec-NhaA and for all mutants that acidify the medium upon Li^+^ binding except EC-NhaA-A167P. This mutant that alkalinizes the medium did not give any signal with Cresol Red. Therefore for the latter mutant we used phenolphthalein which is sensitive at the alkaline range (see below for further details). Changes in proton concentrations upon injection of Li^+^ into the reaction medium were determined spectrophotometrically as described in the “Experimental procedures” section. The absorbance of the indicator Cresol Red (at 577 nm) is highly sensitive to pH changes in the range corresponding to Ec-NhaA activity, pH = 6.5–8.5 (Fig. 5a)^12^. Injection of 30 mM LiCl into the reaction mixture in the absence of the transporter did not produce any change in pH. Figure 5b shows that Li^+^ injection into a sample containing WT Ec-NhaA (15 μM) induced acidification of the medium at a magnitude that was mitigated by adding 35.4 μM KOH. The negative control, the inactive-antiporter mutant Ec-NhaA-D164C, did not show any pH change upon Li^+^ injection (Fig. 5c). Hence, injection of Li^+^ induced proton release from the WT antiporter with a stoichiometry of 2.2 H^+^/Li^+^. A similar experiment with mutant Ec-NhaA-D133C (25 μM) also showed acidification of the medium, but with H^+^/Li^+^ stoichiometry of 4.5 (Fig. 5d). The negative control, D133C-D164N, did not show any pH change upon Li^+^ injection (Fig. 5e).

The mutant Ec-NhaA-A167P in the Cresol Red reaction mixture did not show any change in the medium pH upon Li^+^ injection, implying that this mutant does not acidify the medium upon Li^+^ injection. We therefore used the indicator phenolphthalein with a pH-sensitive range of 8.5–10 (Fig. 5f). Li^+^ injection into the mutant containing Ec-NhaA-A167P (40 μM) and phenolphthalein induced alkalinization of the medium at a level equivalent to 300 μM KOH (Fig. 5g). The negative control mutant A167P-D164N did not show any pH change signal upon Li^+^ injection (Fig. 5h). Hence, the mutant Ec-NhaA-A167P takes up protons with a stoichiometry of 7.5 H^+^/Li^+^. Taken together, the biochemical results fully reproduced the thermodynamic results.

### Circular Dichroism (CD) Spectra of Ec-NhaA Mutants Reflect No Change in Secondary Structures Upon Mutation

To test whether the mutations introduced a major change in the structure of NhaA, we determined the CD spectrum of each protein (Fig. S2). These spectra were very similar to the spectrum of the WT and were typical for membrane proteins containing many helices. These observations indicate that the helical structure of the proteins did not undergo major structural changes. However, neither changes in the conformation of the side chains nor small conformational changes of helices can be identified by the CD spectrum. For this purpose, X-ray crystallography is needed.

## Discussion

Our thermodynamic results for WT Ec-NhaAand mutants (summarized in Fig. 6) show that in all buffers the total enthalpy (Δ*H*_tot_) is larger than the entropy (*T*Δ*S*_tot_), implying that Li^+^ binding to and H^+^ release from Ec-NhaA is enthalpically driven, as we have previously shown^31^. The enthalpic gain is larger than the entropic loss in all cases, and thus the total value of Δ*G*_tot_ is small but still negative, allowing the reactions to proceed. This indicates that upon ion binding and release, favorable interactions occur, resulting in negative enthalpy. The accompanying entropic loss is probably due to local ordering in the protein and binding of multiple ions to it. The overall changes in free energy during Li^+^ binding, Δ*G*_tot_ and Δ*G*_pro*t*_ (Fig. 6), are within the range of −0.2 to−4 kcal/mol (Fig. 6 and Table 1) for a single protein in different buffers (Table 1). The small magnitudes of these changes are consistent with our CD-based observation (Fig. S2) that no major changes in the secondary structure occur as a result of the studied mutations. Furthermore, all conclusions drawn from our thermodynamic experiments were reproduced using direct pH measurements (Fig. 5).

Our detailed thermodynamic analysis, accompanied by biochemical assays of the transport reaction in WT Ec-NhaA and mutants, reveals important insights into the antiport mechanism of Ec-NhaA. These insights are discussed herein.

The Li^+^/H^+^ antiporter mechanism of WT Ec-NhaA is compatible with the canonical alternative access mechanism of secondary transporters^33^. Binding of the Li^+^ and H^+^ ligands to Ec-NhaA is coupled and antagonistic (Figs 2 and 5b), implying that Li^+^ competes with H^+^ for a single binding site that is accessed alternately by each side of the membrane, and these ligands never load the carrier together. Several observations provide evidence that these coupled binding events are part of the physiological transport cycle: The stoichiometry of 2H^+^/Li^+^ obtained here is identical to H^+^: Na^+^ exchange stoichiometry values obtained previously in two different experimental approaches. In one approach, the rates of Na^+^ efflux and proton influx were determined in Ec-NhaA proteoliposomes, and the stoichiometry was calculated from the ratio of the rates^13^. The other approach was based on measuring the membrane potential generated by Ec-NhaA proteoliposomes at various sodium gradients and assuming complete coupling and thermodynamic equilibrium between the membrane potential and the ion^13^. Furthermore, previous electrophysiological results have demonstrated that the transport cycle of Ec-NhaA is associated with displacement of a negative charge across the membrane, as expected for the stoichiometry of 2H^+^/Na^+^ (electrogenicity) of the carrier^27^,^32^, and suggested that Na^+^ competes with H^+^ on the binding site^32^. Nevertheless, the 2H^+^/1Li^+^ stoichiometry found here (Figs 2 and 5b) and in previous studies^13^ for Ec-NhaA is not an absolute value, because there is no way to directly determine the percentage of active/available protein for ion binding, and the ratio does not provide information about the absolute number of bound ions and protons, but only about the ratio between them (i.e., 2:4, 3:6, etc.). Recently, however, a simulation of the Na^+^/H^+^ antiporter activity of Ec-NhaA reached the 2H^+^/1Na^+^ stoichiometry^42^.

Our ITC and pH measurement results together with previous results show that mutations in Asp164 each alter the H^+^/Na^+^, Li^+^ antiporter activity and ligand binding (Li+) or release (H^+^) to an identical extent (Table 1 and S1 and Figs 2 and 5). Thus, Cys-replacement mutations in the active site (D163C and D164C) as shown previously^29^,^30^ and D163E, D163N and D164N as shown before^39^ and here (Table 1), abolish both growth in high Na^+^/Li^+^ selective media and Na^+^/Li^+^ transport activity in everted membrane vesicles and also totally inhibit Li^+^ binding to the protein in all buffers tested (Table 1 and Fig. 2) and abrogate proton release from the proteins (Fig. 5). In contrast, D164E which was shown to grow under high Na^+^ stress conditions (pH 7) and to possess a significant antiport activity^39^ (Table S1) showed Li^+^-coupled H^+^ release with a 2H^+^/Li^+^ stoichiometry similar to that of the WT but with a lower affinity (4–6-fold higher *K*d).

These results support the notion that, at physiological pH, Asp164 is protonated^43^, and that upon Li^+^ binding it acts as a proton donor and releases an H^+^. This conclusion is consistent with several observations that have been obtained previously, using different approaches: i) Asp164 is one of the most evolutionarily-conserved residues^44^. ii) It is an essential residue for growth under high Na^+^/Li^+^ selective media^29^,^45^. iii) In the two available Ec-NhaA crystal structures of the inward-open conformation^18^,^21^, Asp164 is located at the bottom of the cytoplasmic funnel exposed to the cytoplasm^18^,^21^ (Fig. 1a) and is potentially exposed to non-hydrated Na^+^ or Li^+ 18^. iv) Computations based on either Ec-NhaA crystal structure all predict that Asp164 is protonated^21^,^46^,^47^ and releases its proton upon Na^+^ binding.

Like its adjacent neighbor, Asp163, located on TM V (Fig. 1), is a highly evolutionarily-conserved residue. It is essential for growth under high Na^+^/Li^+^ selective media, and in both available Ec-NhaA crystal structures it is buried in the protein and not exposed to the cytoplasmic funnel^18^,^21^ (Fig. 1). Yet, the neighborhood of Asp163 differs between the two crystal structures, due to a difference in the position of Lys300. Whereas in the newer crystal structure, Asp163 forms a salt bridge with Lys300 of helix X^21^ (Fig. 1), in the original crystal structure Lys300 is about one turn of a helix away from Asp163^18^. It is still debatable whether the two structures represent two conformations, or whether TM X was mis-assigned in the original structure determination. Computations on the basis of the original crystal structure have predicted that both Asp163 and Asp164, are protonated at alkaline pH, bind Na^+^ and release H^+^ 46,47. In marked contrast, molecular dynamics simulations based on the new structure predict that although the origin of one proton is the protonated Asp164, as described above, the other proton comes from the protonated Lys300 forming the salt bridge with D163.When Na^+^ enters the funnel, Lys300 releases the second H^+^ (and leaves Asp163), and Na^+^ binds to both Asp163 and Asp164. Nevertheless, the authors^21^ do not rule out the possibility that Asp163 can transiently be a proton donor. Our ITC and biochemical results with Asp163 mutations are compatible with both options, because the tested mutations, D163C/N/E abrogated Na^+^/Li^+^ transport^39^ (Table S1) as well as Li^+^ binding to or H^+^ release from the antiporter (Figs 2f and 5c).

**Figure 3:**
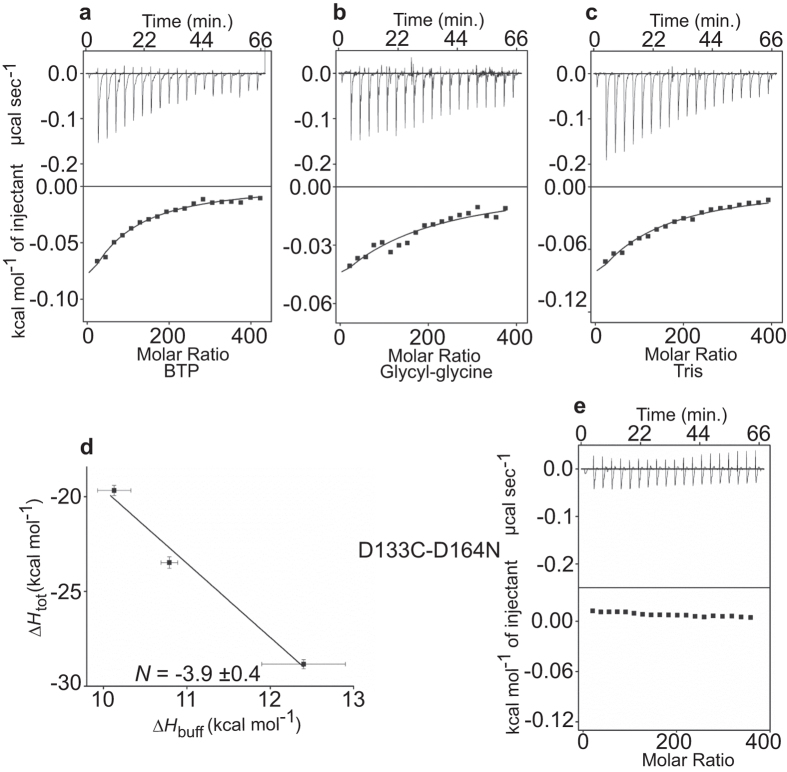
The Ec-NhaA-D133C mutation changes the stoichiometry of Li^+^/H^+^ binding. The buffers in panels (**a,b**) are as in Fig. 2, and in panel (**c**) the buffer is Tris. Each reaction chamber contained Ec-NhaA-D133C. (Averaged thermodynamic parameter values are reported in Table 1.) (**d**) Plot of the total enthalpy, Δ*H*_tot_, of Li^+^ binding as a function of the enthalpy of buffer ionization, Δ*H*_buff_. Squares, experimental data. (**e**) An example of an experiment done in BTP buffer with mutant Ec-NhaA-D133C-D164N. The errors for the experimental points are the s.e.m.

The ITC (Fig. 3) and pH measurement (Fig. 5d) results of the mutant D133C are consistent with previous results suggesting that D133 is in the H^+^ pathway and possibly in the active site^31^. Asp133 is a highly evolutionarily-conserved residue and its mutation D133C produces antiport activity with high apparent *K*m for Li^+^\Na^+^ 30 (Table S1). The mutant protein binds Li^+^ 31, and at the same time, as shown here, it antagonistically releases H^+^ with a stoichiometry of 4H^+^/1Li^+^ (Figs 3 and 5d) as opposed to 2H^+^/1Li^+^ of the WT (Figs 2 and 5b), and with an increased *K*d (Figs 3 and 5d, Table 1). As discussed above, the stoichiometry is not an absolute value, a mutant may change the value of the slope not because it changes the stoichiometry but because it changes how much of the protein sample is protonated at the beginning of the Li^+^ titration in the ITC experiment.

Nevertheless, the increased *T*Δ*S*_tot_ in D133C (Fig. 6) correlates with the increased *K*d for Li^+^ binding and for the change in stoichiometry of the mutant (Figs 3 and 5d). This change may relate to the strategic location of Asp133 in the extended chain (TM IV) of Ec-NhaA in the middle of the membrane, where it balances a delicate electrostatic interaction in the TM IV/XI assembly^18^ (Fig. 1). In addition, unlike in a helix, the extended chain itself is not saturated by hydrogen bonds and can donate main chain carbonyl to coordinate the cation as part of the binding site^31^. Hence, a mutation like D133C can change the cation interactions.

The enthalpic changes that occur to the antiporter protein upon Li^+^ binding and H^+^ release, Δ*H*_prot_, may be associated with ion binding and concomitant release by the protein, as well as rearrangement of the protein structure^42^ and the surrounding solution. Here, these values show a similarity between the WT and the mutants D164E and D133C, all of which display a positive value for Δ*H*_prot_ on the order of 10–20 kcal/mol (Fig. 6 right and Table 1b). This value is mitigated by the enthalpic contributions of ion binding to the buffer (Δ*H*_buff_), so that the total enthalpy change, Δ*H*_tot_ = Δ*H*_prot_^+^ *N*Δ*H*_buff_ < 0 (Fig. 6 left). We conclude that the buffer-associated enthalpic changes dominate the overall free-energy associated with transporter action. Thus, the thermodynamic analysis shows that not only the protein but also the cellular environment (proton acceptors) plays an important role in the transport mechanism.

**Figure 4:**
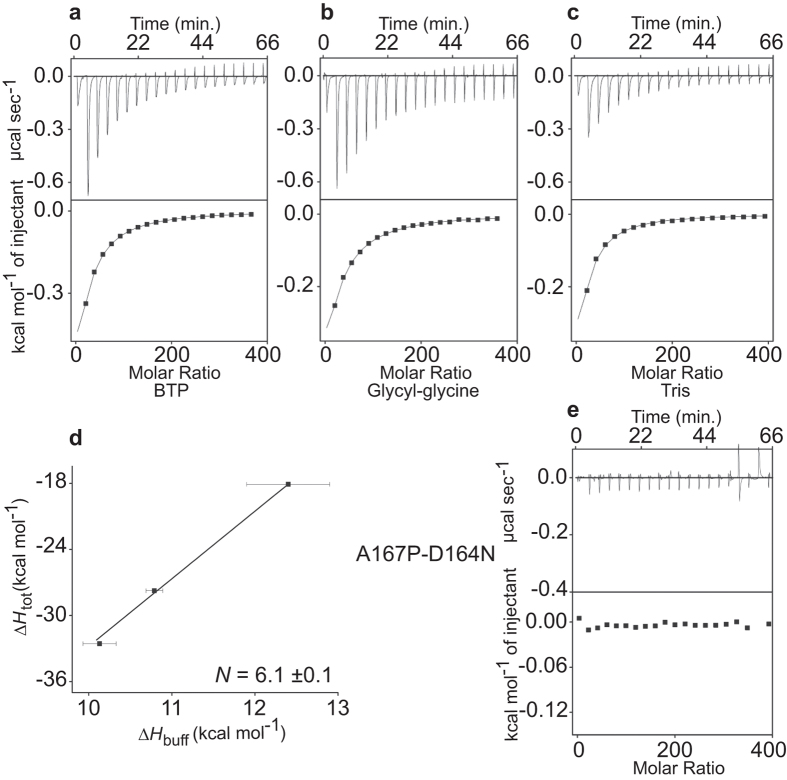
Synergistic binding of Li^+^ and H^+^ to Ec-NhaA-A167P. Panels (**a–c**) as in Fig. 3 but containing the mutant Ec-NhaA-A167P. (Averaged thermodynamic parameter values are reported in Table 1). (**d**) Plot of the total enthalpy, Δ*H*_tot_, of Li^+^ binding as a function of the enthalpy of buffer ionization, Δ*H*_buff_. Squares, experimental data. The errors for the experimental points are the s.e.m. (**e**) Examples of the ITC data obtained with the double mutant A167P-D164N.

**Figure 5:**
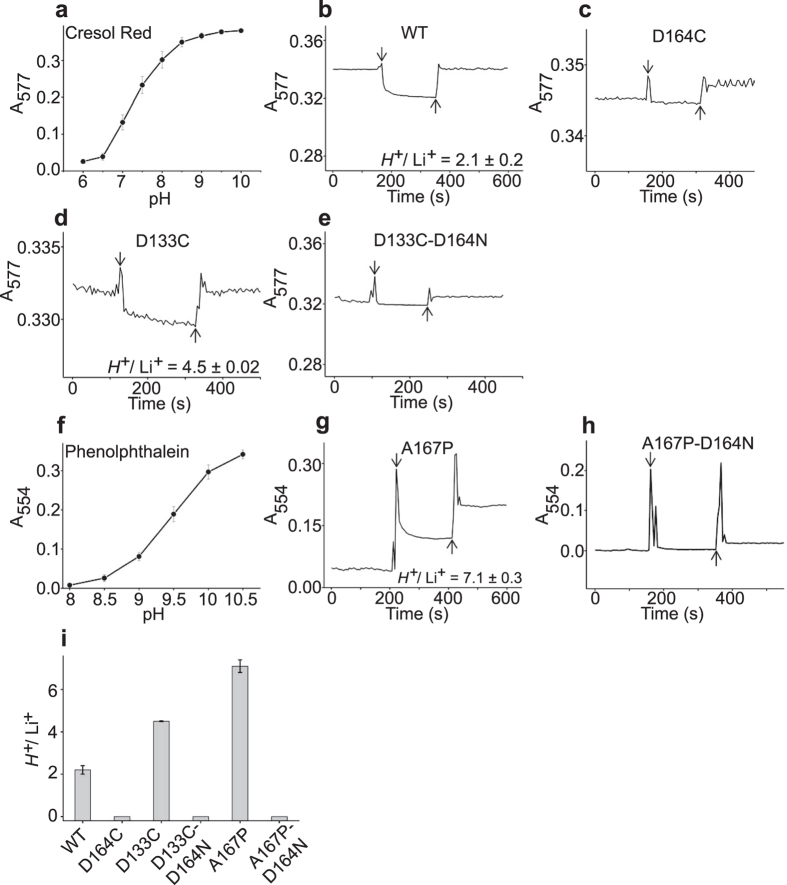
Li^+^-Induced proton release or uptake of Ec-NhaA variants as measured by Li^+^- induced absorbance changes of pH indicators in the reaction medium. (**a–h**) All reaction media were prepared at pH 8.5 as described in “Experimental Procedures”, contained unbuffered 100 mM KCl, the respective pH indicator and proteins as indicated, except reaction media (**a,f**), which were prepared without protein to determine the pH sensitivity of the indicators in the reaction medium. Each experiment was repeated at least 3 times with practically identical results. (**a**) Cresol Red (100 μM) has a pH sensitivity in the range of pH 6.5–8.5, which is the pH range of activity of the antiporter^12^. Therefore, it was suitable for monitoring Li^+^-induced acidification of the medium caused by all Ec-NhaA variants except Ec-NhaA-A167P. (**b**) WT Ec-NhaA. The reaction solution contained 15 μM purified WT Ec-NhaA. After steady-state absorbance was reached, Li^+^ (30 mM) was injected (downward-facing arrow), resulting in acidification of the medium. Further addition of Li^+^ did not release additional protons, indicating that the effect was saturable. Then, 20–300 μM KOH was injected (upward-facing arrow) to determine the amount of hydroxyl equivalents needed to cancel the acidification signal. All other experiments were conducted in a similar manner. (**c**) Ec-NhaA-D164C (25 μM), the antiporter-inactive variant of Ec-NhaA, a negative control that did not show any pH change upon injection of Li^+^. (**d**) Mutant Ec-NhaA-D133C (25 μM). (**e**) D133C-D164N (30 μM), an inactive antiporter, the negative control of D133C. (**f**) Phenolphthalein (63 μM) has a pH sensitivity in the range of pH 8.5–10. Therefore, it was suitable for monitoring Li^+^-induced alkalinization of the medium caused by the mutant Ec-NhaA-A167P. (**g**) Mutant Ec-NhaA-A167P (40 μM). (**h**) Ec-NhaA-A167P-D164N (35 μM), an inactive antiporter, a negative control of Ec-NhaA-A167P. (**i**) Summary of H^+^/ Li^+^ stoichiometry of Ec-NhaA and its variants normalized to protein concentration with the error bars (s.e.m.) shown.

**Figure 6:**
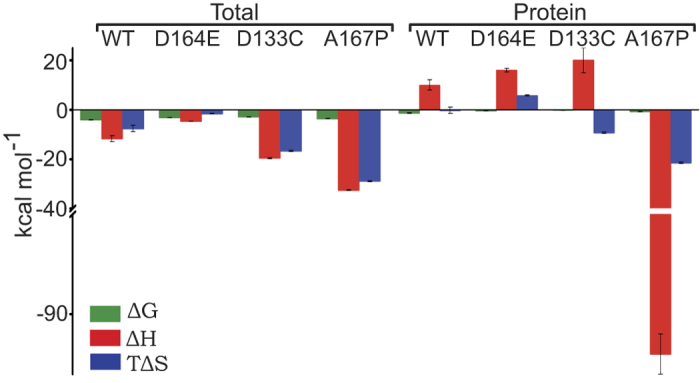
The total and the protein-specific enthalpic and entropic contributions to the Gibbs free energy for Li^+^ binding to Ec-NhaA variants. The values presented were obtained in the BTP reaction mixtures (Table 1).

Striking in its dissimilarity to the WT and mutants discussed above is A167P: 1) ITC experiments show that, in contrast to the antagonistic binding of 2H^+^/Li^+^ in WT Ec-NhaA (Fig. 2 and Table 1), in Ec-NhaA-A167P H^+^ and Li^+^ binding is synergistic (Fig. 4), with H^+^/Li^+^ stoichiometry of about 6. 2) Identical results were obtained by measuring the proton uptake from the medium (compare Fig. 5g to Fig. 5b,d). 3) Our previous combined biochemical and electrophysiological study^41^ has shown that this mutant is electrogenic^41^ and the mutation changes the rate-limiting step of the Ec-NhaA turnover cycle^41^; A167P possesses the electrogenic reaction of the Na^+^/ Li^+^-loaded carrier observed in the WT, but the rate limiting step of the mutant is an electroneutral reaction of the proton-loaded carrier. 4) Because the CD spectrum of the mutant A167P is very similar to that of the WT (Fig. S2), it appears that the mutation does not drastically affect the helicity of the mutated protein. However, a proline replacement is expected^48^ to introduce a conformational change at least in the side chain, which may be reflected in other side chains and possibly also in the protein’s accessibility to water. 5) Ec-NhaA-A167P displays a much higher negative enthalpic change upon Li^+^ binding, Δ*H*_prot_ = −94 ± 2 kcal/mol (Fig. 6). This not only highlights the stark difference between A167P, which displays synergistic Li^+^/H^+^ binding, and the other mutants, which display antagonistic binding, but also hints at the possibility of new bonds or structural rearrangements in the mutant transporter dynamics.

It is possible to argue that the behavior of the purified A167P protein does not reflect its transport mechanism in the membrane. However, our previous combined biochemical and electrophysiological study^41^ has shown that A167P displays transport activity with a high apparent *K*m both for Na^+^ and for Li^+^ in isolated everted vesicles and in proteoliposomes, and, similarly to the WT, is electrogenic.

Based on the NhaA crystal structures obtained at acidic pH, we explored possible structural determinants that would allow us to recognize H^+/^binding sites in A167P protein. This was a challenging task since no obvious conserved motifs for H^+^ or Na^+^ binding site have been established yet. While H^+^ binding requires the presence of hydrophilic groups, Na^+^ coordination requires several ligands with an appropriate spatial geometry. In some enzymes, subtle differences in just one specific amino acid residue seem to define the enzyme specificity for Na^+^ or H^+^ ions. In WT NhaA as shown here, negatively charged residues (D133, D163, D164) positioned in the transmembrane portions of the protein comprise the Li^+^ binding site which exchanges for H^+^. We suggest that similar residues exist in A167P and fulfill similar role. Nevertheless, the presence of net negative charges is not a strict requirement neither for Na^+^ transport nor for H^+^ binding. Other polar amino acid residues around the Li binding site (T132, S341) can participate in extra H^+^ binding. Furthermore, the binding site in NhaA is at the crossing of the two extended chaines. Because these are flexible and not saturated by hydrogen bonds they can contribute backbone carbonyl groups for H^+^ binding. In addition computation showed that water molecules can penetrate this area upon Li^+^ binding.

**Figure 7:**
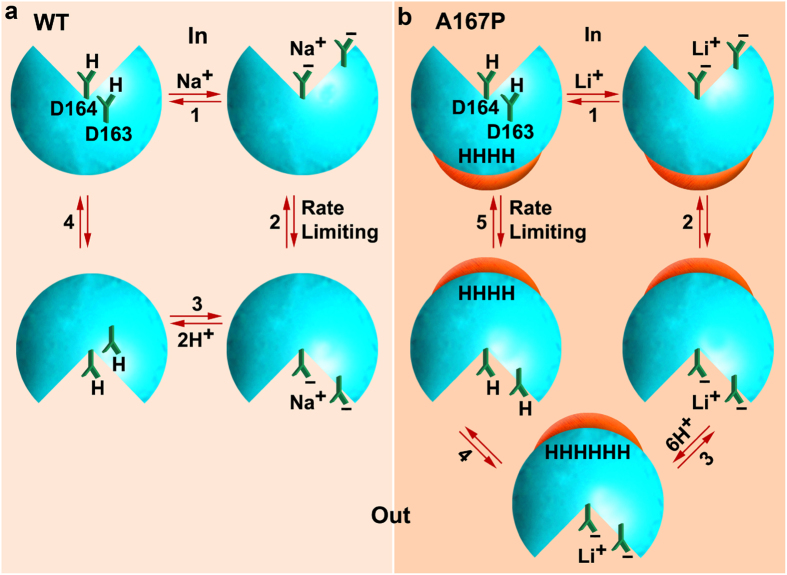
A schematic representation of the reaction cycles of the WT Ec-NhaA and mutant EC-NhaA-A167P. (**a**) WT Ec-NhaA, the canonical alternate access scheme for antagonistic 2H^+^/Li^+^ antiport. (**b**) Mutant Ec-NhaA-A167P shows synergistic Li^+^/ 6H^+^ binding. For details see text.

However, the available crystal structures of NhaA were obtained at pH 4^2^ or 3.5^3^ when NhaA was down regulated (inactive). Therefore, speculations on the basis of these structures may lead to mistakes. Hence, Fig. 7 summarizes the overall input of the ITC and biochemical results of our manuscript.

On the basis of electrophysiological data^32^, ITC data (Figs 2 and 4) and biochemical data (Fig. 5b,g), we suggest the following schematic models for the turnover cycles of WT Ec-NhaA (Fig. 7a) and mutant Ec-NhaA-A167P (Fig. 7b) at alkaline pH when both are active (Table S1): (1) Both in WT and in the A167P mutant, the protonated Asp164 and Asp163 at the cytoplasmic side deprotonate and bind cytoplasmic Na^+^/Li^+^, which only partly compensates for their negative charges. The mutant also deprotonates the extra H^+^. (2) A conformational transition of the Na^+^/Li^+^-loaded carrier outward is thus associated with displacement of a net negative charge across the membrane, an electrogenic step that is rate-limiting in the WT but not in the mutant^32^,^41^. (3) In the WT, Na^+^/Li^+^ is released outside, while two protons antagonistically bind the aspartates, fully compensating for their charges. In contrast, in the mutant, the Li^+^-loaded carrier induces binding of 6 protons. (4) In the WT, a conformational transition inward of the 2H^+^-loaded WT carrier, an electroneutral reaction, re-starts the cycle. In contrast, the mutant, loaded with 6 protons and 1Li^+^, changes conformation, which releases the Li^+^, and two protons protonate the aspartates. (5) The 6-H^+^ loaded carrier moves inward to complete the cycle. This is the rate-limiting step of the mutant, as shown by electrophysiology^41^. Clearly, more experiments are needed to support this working hypothesis. Furthermore, it will be most interesting to determine the crystal- structure of mutant Ec-Nha-A167P.

The CLC protein family comprises two sub-classes of membrane transport proteins: Cl^−^ channels and H^+^/Cl^−^ exchangers. All CLCs are homodimers, with each monomer forming an individual Cl^−^ permeation pathway, which appears to be largely similar between the two CLC sub-classes^49^. Previous ITC measurements^34^ conducted with Ec1-CLC have shown that this anionic antiporter is very different from Ec-NhaA, the cationic antiporter: In contrast to WT Ec-NhaA, which obeys the canonical alternative access mechanism and binds Li^+^/H^+^ antagonistically (Fig. 2)^33^ (see above), chloride binding to Ec1-CLC induces synergistic protonation of a crucial glutamate and the simultaneous binding of 1H^+^ and 2Cl^−^ gives rise to a fully-loaded state that is incompatible with conventional transport mechanisms. Mutations in the Cl^−^ transport pathway alter the stoichiometries of H^+^/Cl^−^ exchange and binding to an identical extent, and it has been proposed that the thermodynamics of synergistic substrate binding, rather than the kinetics of conformational changes and ion binding, determine the stoichiometry of transport^34^. Surprisingly, like Ec1-CLC, mutant Ec-NhaA-A167P also shows synergistic binding of protons, although with a stoichiometry of 1Li^+^/6H^+^ (Figs 4 and 5g).

Both antiporters Ec-NhaA and Ec1-CLC are highly important for ion homeostasis of the cell, and their homologues are drug targets; however, the two proteins are evolutionarily distant from each other^18^ and differ in crystal structure^18^,^50^. Ec1-CLC belongs to a family that contains many channels^34^,^49^, and Ec-NhaA belongs to a very specific part of the Cation Proton Antiporter superfamily (CPA)^28^. Further structural/functional research is needed to address how different structures, and even a single mutation in one structure, as shown here, can adapt to different substrates and different substrate/proton stochiometries and mechanism.

## Methods

### Plasmid, Bacterial Strains, and Culture Conditions

TA16 (*mel*BLid, Δ*lac*ZY, *thr*1, and *lac*IQ^12^ and EP432 (*mel*BLid, Δ*nha*A1::*kan*, Δ*nha*B1::*cat*, Δ*lac*ZY, and *thr*1^51^) are *E. coli* K-12 derivatives. Cells were grown either in Luria broth (LB) or in modified Luria broth in which NaCl was replaced with KCl (LBK^52^). The medium was buffered with 60 mM 1,3-Bis[tris(hydroxymethyl)methylamino]propane (BTP), and 100 μg/ml ampicillin was added. For plates, 1.5% agar was used.

To test cell resistance to Li^+^ and Na^+^, EP432 cells transformed with the respective plasmids were grown on LBK to *A*_600_ of 0.5. Samples (2 μl) of serial 10-fold dilutions of the cultures were spotted onto agar plates containing the selective media: modified LB in which NaCl was replaced with the indicated concentrations of NaCl or LiCl at the various pH levels and incubated for 2 days at 37 °C.

Plasmids pAXH^53^ and pAXH3^54^ are pET20b (Novagen) derivatives encoding His-tagged NhaA. pAXH3 lacks the BglII site at position 3382^30^ and contains a BstXI silent site at position 248 in *nha*A^55^. All plasmids carrying mutations are designated by the name of the plasmid followed by the mutation. The plasmids pAXH-D163C, pAXH-D164C, and pAXH-D133^45^ and pAXH-T132C^30^ encode the NhaA mutations D163C, D164C, D133C, and T132C, respectively. The plasmids pAXH-D163E, pAXH-D163N, D164E and D164N encode the variants D163E, D163N, D164E and D164N, respectively^31^,^39^.

### Site-Directed Mutagenesis

Site-directed mutagenesis was conducted following previously published protocols^56^, with pAXH3 as a template. Mutants D133C-D164N and A167P-D164N were constructed and their DNA sequences were verified.

### Overexpression and Purification of NhaA Protein Variants

Overexpression of the NhaA variants^57^ and affinity purification (Ni^2+^-nitrilotriacetic acid-agarose, Qiagen)^58^ were performed as described previously, but the protein was eluted in a buffer (pH 7.9) containing 300 mM imidazole, 25 mM citric acid, 100 mMKCl, 5 mM MgCl_2_, and 0.015% *n*-dodecyl β-D-maltopyranoside (DDM). After the addition of sucrose (10%) to the eluted protein solution, the protein solution was dialyzed overnight at 4 °C in acidic elution buffer^53^ containing 10% sucrose, 100 mMKCl, 25 mM citric acid, 5 mM MgCl_2_, 0.015% DDM (pH 4.0) and was then frozen at−80 °C.

### Detection and Quantification of NhaA Variants in the Membrane

Total membrane protein was determined using the Bradford assay^59^. The expression levels of His-tagged NhaA variants were determined by resolving the Ni^2+^-nitrilotriacetic acid, purifying the proteins on SDS-PAGE, staining the gels with Coomassie Blue staining, and quantifying the band densities using Image Gauge (Fuji) software^53^.

### Isothermal Titration Calorimetry (ITC)

The NhaA variants were prepared for ITC as previously described^31^ by thawing each frozen protein (about 1.2 ml solution containing ~600 μg protein) at 4 °C, concentrating it 4-fold by filtration (Amicon Ultra, 30 K) and washing 3 times (with the original volume) in one of the 4 different reaction buffers containing 50 mM BTP or 2-Amino-2-hydroxymethyl-propane-1,3-diol (Tris) or glycyl-glycine or N-[Tris(hydroxymethyl)methyl]-3-aminopropanesulfonicacid,[(2-Hydroxy-1,1-bis(hydroxymethyl)ethyl)amino]-1-propanesulfonic acid (TAPS), 150 mM choline chloride, 5 mM MgCl_2_, 10% sucrose (pH 8.5). These washing cycles yielded a reaction mixture for ITC, containing 20–25 μM of the NhaA protein and 0.06–0.08% DDM.

ITC experiments were performed using the micro-calorimeter ITC200 (MicroCal, GE Healthcare). A reaction mixture for ITC (300 μl) of WT Ec-NhaA or its variants was loaded into the sample cell. For titration, 40 mM LiCl, dissolved in the ITC reaction buffer, were loaded into the injection syringe. Before data collection, the system was equilibrated to 10 °C with the stirring speed set to 500 r.p.m. Titration curves for binding Li^+^ were initiated by an injection of 0.8 μl followed by successive 2-μl injections of the ligand every 200 s. Injections of ligands into the reaction buffer without protein, in addition to injections of reaction buffer into reaction buffer, were performed as controls to determine background corrections. The integrated heats from each injection, normalized to the moles of ligand per injection, were fit to a single-site binding isotherm using Origin 7 software. The integrated peak of the first injection was excluded from the fit due to the large errors in the first step. To minimize the potential for artifacts in our binding assays arising from contaminating Na^+^ co-purified with NhaA, we washed the protein extensively in the reaction buffer, which is practically Na^+^-free (<1 μM Na^+^ as determined by atomic absorption).

### Fitting ITC Data

Fits were performed as described previously^38^. The stoichiometry of the Li^+^ binding to NhaA and its mutants was fixed to 1:1 for the fit. It could not be determined experimentally by ITC due to the very high Kd values (in the milimolar range). This results in a C value lower than 1, since the protein concentration used experimentally is lower than Kd. Higher protein concentrations (in the milimollar range) cannot be reached because the protein aggregates at these concentrations. The data were fit to the Wiseman isotherm using the Origin ITC analysis package. The different shapes of the ITC curves are due to the different affinities of Li^+^ for WT and mutant Ec-NhaA variants. The general shape of an ITC curve is determined by the value of the product of the association constant and the protein concentration (the c-value). When c > 5, the curve takes a characteristic sigmoid shape; when c < 5 the curve becomes progressively more hyperbolic for lower values of c. For WT Ec-NhaA, c < 1 in all buffers, so the curves are hyperbolic. Similarly, other Ec-NhaA mutants showed hyperbolic curves (c < 1), corresponding to lower affinity for Li^+^. The enthalpy of ionization of the various buffers, Δ*H*_buff_, was determined experimentally (Fig. S1) following established protocols^60^.

### Measurement of Li^+^-Induced pH Change of the Medium Using pH Indicators

To control the pH of the media of these experiments, it was essential to degas all solutions, as atmospheric CO_2_ can cause changes to solution pH. Therefore, each solution used was prepared in gas-tight sealed vessels, and was degassed for 0.5 h by vacuum suction. Next, argon was blown through the solution for 0.5 h. The affinity-purified Ec-NhaA variant proteins (15–40 μM) were washed three times in 2 ml of unbuffered 0.1 M KCl on Amicon ultra centrifugal filters (Ultracel 30 k, Merck, Darmstadt, Germany). All proteins, with the exception of Ec-NhaA-A167P, were then resuspended in 400 μl containing 0.04–0.06% DDM and 100 μM Cresol Red (Sigma); for Ec-NhaA-A167P, 63 μM phenolphthalein (Mallinckrodt Chemical Inc., St. Louis, MO) was used instead of Cresol Red. Because pH 8.5 was the starting pH, the pH range of Cresol Red suited acidification of the medium, whereas phenolphthalein suited alkalinazation of the medium upon Li^+^ injection. Before the experiment, each solution was brought to pH 8.5 (as measured by microelectrode, Elhamma, Israel) using 50 mM KOH, and was introduced into a glass cuvette (of 1 ml with a cover) and overlaid by 100 μl liquid paraffin. The pH of the medium was measured spectroscopically in a diode-array UV-visible spectrophotometer (Agilent 8453), recorded at 577 nm for Cresol Red or at 554 nm for phenolphthalein, at 25 °C. After a stable pH (absorbance) was reached, the experiment was started by injecting 30 mMLiCl with a gas-tight glass syringe (Hamilton, 25 μl). The solution was mixed by introducing a needle (size 19) of a glass syringe (2 ml) into the cuvette’s bottom and rapidly sucking in and pushing back about half of the solution volume two times. This mixing, without previous addition of Li^+^, did not change the reaction medium’s pH and was used whenever mixing was needed. Down or upward deflection of absorbance reflects acidification or alkalinization of the medium, respectively. For each experiment, a negative control of a corresponding transport-inactive variant was run in parallel to make sure that the change in pH reflected Li^+^-induced proton uptake or release by the protein. To quantify the protons that were released or up-taken by each Ec-NhaA variant, we determined the amount of 20–200 μM KOH that had to be added to cancel the change in pH.

## Additional Information

**How to cite this article**: Manish, D. *et al.* The Ec-NhaA antiporter switches from antagonistic to synergistic antiport upon a single point mutation. *Sci. Rep.* **6**, 23339; doi: 10.1038/srep23339 (2016).

## Supplementary Material

Supplementary Information

## Figures and Tables

**Table 1:** Thermodynamic parameters for Li^+^ binding to WT NhaA and mutants.

a							
	Buffer	*N*(H^+^/Li^+^)	∆*H*_tot_ (kcal mol^−1^)	∆*S*(cal mol^−1^ deg^−1^)	*T*∆*S*_tot_ (kcal mol^−1^)	∆*G*_tot_ (kcal mol^−1^)	Kd (mM)
WT	BTP	−2.1 ± 0.1	−11.8 ± 1.2	−27.2	−7.7 ± 1.3	−4.1 ± 0.1	0.9 ± 0.1
	Glycyl-glycine		−12.8 ± 0.6	−32.2	−9.1 ± 0.6	−3.7 ± 0.1	1.3 ± 0.2
	TAPS		−13.5 ± 0.4	−34.2	−9.9 ± 0.4	−3.8 ± 0.01	1.1 ± 0.01
	Tris		−16.5 ± 0.6	−45.0	−12.7 ± 0.6	−3.8 ± 0.1	1.2 ± 0.3
D164E	BTP	−2.08 ± 0.1	−4.7 ± 0.03	−5.2	−1.6 ± 0.1	−3.1 ± 0.04	3.5 ± .1
	Glycyl-glycine		−6.1 ± 0.2	−9.8	−2.8 ± 0.3	−3.3 ± 0.1	2.8 ± 0.3
	Tris		−9.2 ± 1.2	−21.8	−6.2 ± 1.4	−3 ± 0.2	4.9 ± 1.6
D133C	BTP	−3.9 ± 0.4	−19.7 ± 0.2	−59.2	−16.8 ± 0.3	−2.9 ± 0.1	5.8 ± 1.3
	Glycyl-glycine		−23.5 ± .3	−72.8	−20.6 ± 0.8	−2.8 ± 0.3	6.8 ± 1.4
	Tris		−28.8 ± 0.2	−92.0	−26.1 ± 0.3	−2.7 ± 0.04	7.1 ± 0.2
A167P	BTP	6.1 ± 0.1	−32.6 ± 0.1	−105	−29.0 ± 0.2	−3.6 ± 0.02	1.7 ± 0.1
	Glycyl-glycine		−27.7 ± 0.05	−85.3	−24.2 ± 0.1	−3.5 ± 0.04	1.7 ± 0.1
	Tris		−18.1 ± 0.01	−50.7	−14.4 ± 0.01	−3.7 ± 0.1	1.3 ± 0.1
b							
	*ΔH*_prot_ (kcal mol^−1^)		*ΔG*_prot_ (kcal mol^−1^)		*TΔS*_prot_ (kcal mol^−1^)		
WT	10 ± 2		−1.4 ± 0.1		−0.3 ± 1.1		
D164E	16 ± 0.6		−0.4 ± 0.06		5.8 ± 0.2		
D133C	20 ± 5		−0.2 ± 0.1		−9.4 ± 0.3		
A167P	−94 ± 2		−0.8 ± 0.05		−21.6 ± 0.3		

(a) Total thermodynamic values Kd and ΔH_tot_ were obtained from a fit to a binding isotherm, and free energy difference (ΔG) and entropy (T∆S) were calculated from equation 2, where R is the molar gas constant and T is the absolute temperature. We fixed the number of Li^+^ at 1. In all cases, Δ*H*_tot_ represents the total enthalpy generated during the binding reaction. All experiments were repeated at least three times. Values are reported as the mean ± s.e.m. of the independent experiments. (b) Protein-specific thermodynamic values.
