# Supplementary material for: The Ec-NhaA antiporter switches from antagonistic to synergistic antiport upon a single point mutation
Source: Sci Rep. 2016 Mar 29;6:23339. doi: 10.1038/srep23339 (PMC4810432; doi:10.1038/srep23339)
Supplement: Supplementary Information [file srep23339-s1.doc]

**The Ec-NhaA transporter switches from antagonistic to synergistic antiporting upon a single point mutation**

**Dwivedi Manish, Sukenik Shahar, Friedler Assaf and Padan Etana**

| **Mutant** | **Expr-**  **ession** | **Growth**  **pH 7** | | **Transport activity in membrane**  **% WT** | | **Apparent *K*m (mM)** | | **Ref.** |
| --- | --- | --- | --- | --- | --- | --- | --- | --- |
|  | **% WT** | **Na^+^**  **0.6 M** | **Li^+^**  **0.1 M** | **Na^+^** | **Li^+^** | **Na^+^** | **Li^+^** |  |
| D163C | 98 | - | - | - | - |  |  |  |
| D163N | 23 | - | - | - | - |  |  |  |
| D163E | 41 | - | - | - | - |  |  |  |
| D164C | 99 | - | - | - | - |  |  |  |
| D164N | 100 | - | - | - | - |  |  |  |
| D164E | 100 | + | - | 39 | 65 | 67 | 3 |  |
| D133C | 93 | + | + | 87 | 70 | 3.6 | 1.24 |  |
| D133C-D164N | 80 | - | - |  |  |  |  |  |
| A167P | 70 | - | +++ | 55 | 48 | 1.2 | 0.4 |  |
| A167P-D164N | 90 | - | - |  |  |  |  |  |
| WT | 100 | +++ | +++ | 100 | 100 | 0.2 | 0.02 |  |

**Supplementary Information:**

**Table S1. Functional characteristics of Ec-NhaA variants.** Mutants A167P-D164N and D133C-D164N were constructed in this work. The data of the other mutants were collected from the indicated references. -, no growth; +++, growth similar to the WT; +, number of colonies similar to the WT but with smaller size.


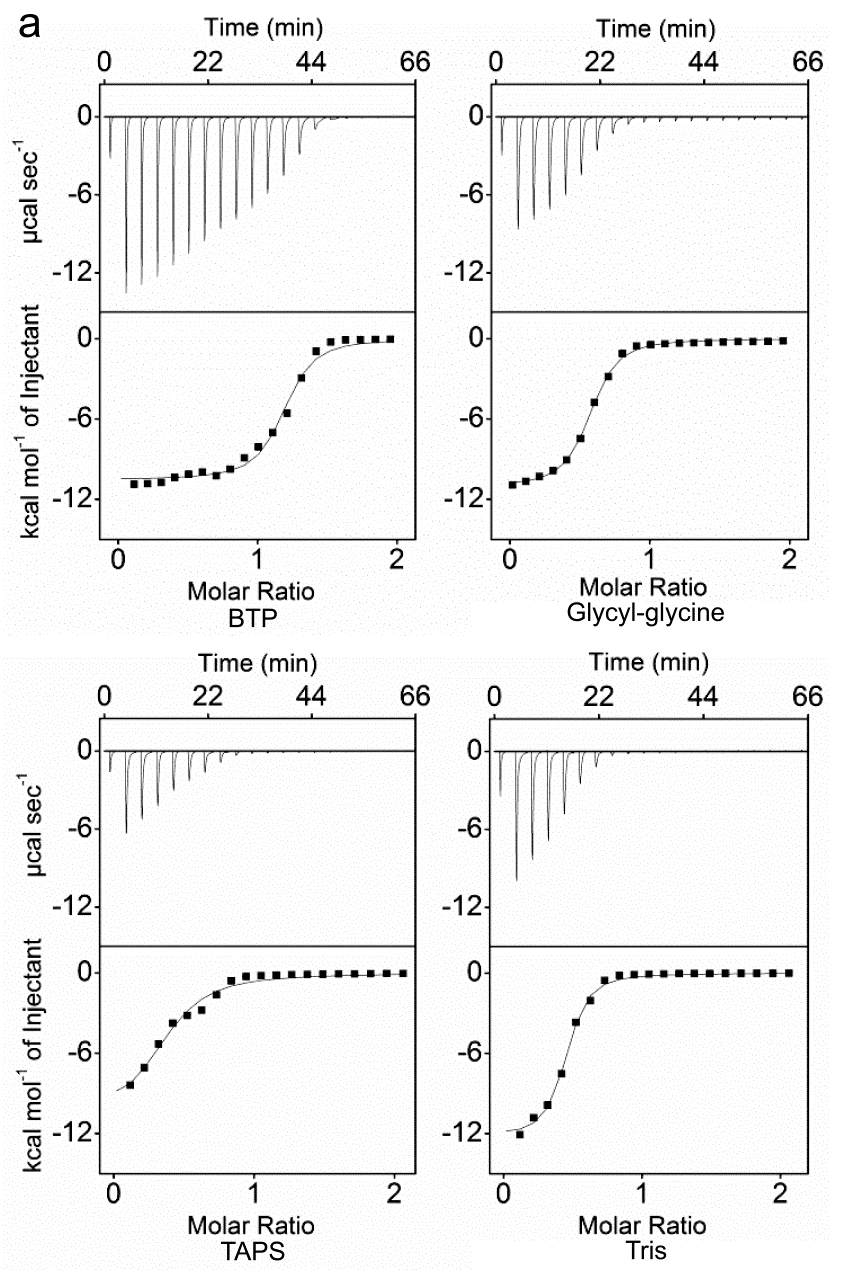
 **b.**

| **Buffer** | **∆*H*_buff_**  **(kcal mol^-1^)** | **pKa** |
| --- | --- | --- |
| BTP | 10.1 ±0.2 | 9.0 |
| Glycyl-glycine | 10.8 ±0.1 | 8.4 |
| TAPS | 11.0 ±0.1 | 8.4 |
| Tris | 12.4 ±0.5 | 8.3 |

**Figure S1. Experimental determination of buffer ionization.**

a. Buffer ionization. The enthalpy of buffer ionization was measured using ITC following published protocol^4^. Briefly, a solution containing 150 mM choline chloride, 5 mM MgCl_2_, 10 % sucrose and 10 mM HCl was injected into the experimental chamber which contained 150 mM choline chloride, 5 mM MgCl_2_, 10 % sucrose, 1 mM BTP or Glycyl-glycine or TAPS or Tris, at pH 8.5. Each experiment consisted of 20 injection of 2 µl of the injection solution at 2-4 min intervals. The enthalpy of buffer ionization was determined by fitting the data to the Wiseman isotherm. b. Δ*H*_buff_ and pKa of the buffers.

**
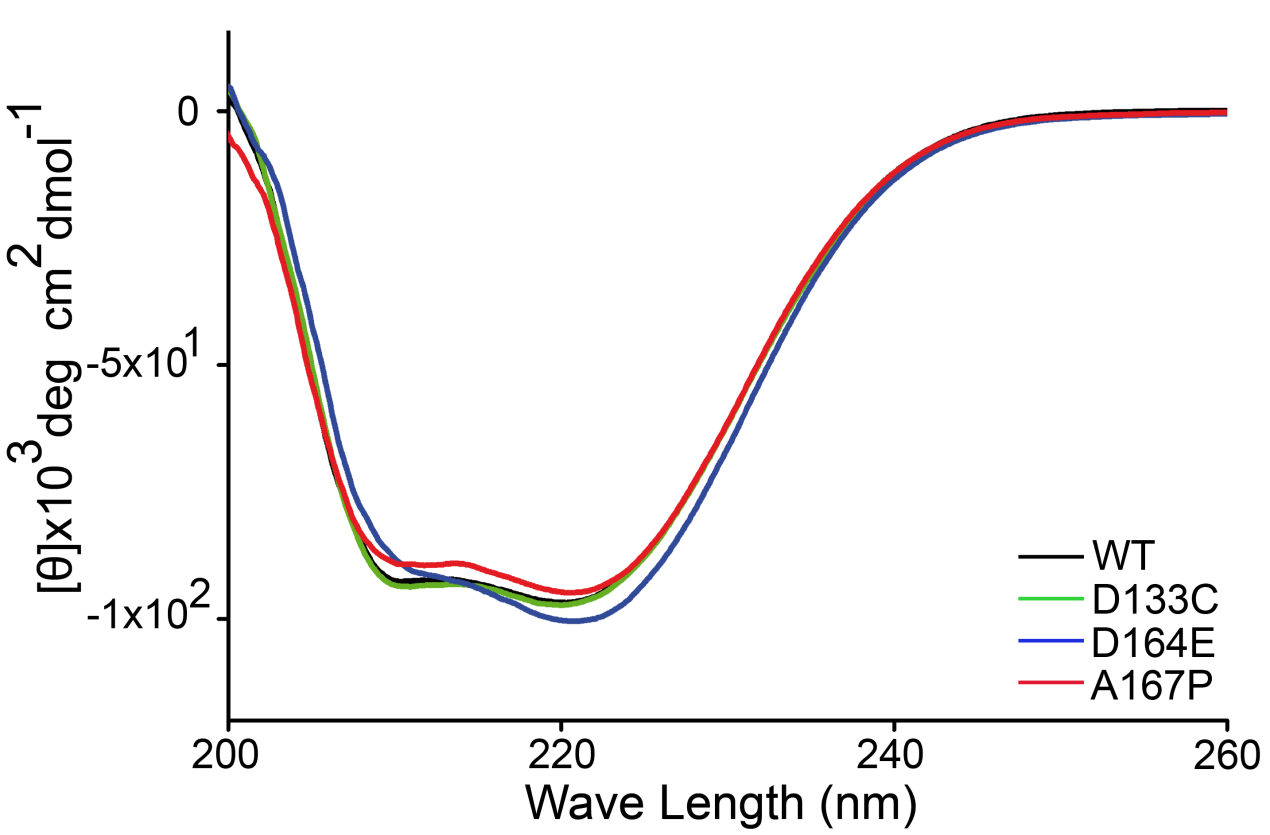
**

**Figure S2. CD spectra of WT Ec-NhaA and mutants.** The respective proteins (6-8 µM) were prepared in 20 mMTris reaction buffer at pH 8.5, and the CD spectra were recorded on a JASCO J-810 CD Spectro-polarimeter (JASCO, Inc., Japan), using the supplied Spectra Manager software.

**References**

1. Galili L, Rothman A, Kozachkov L, Rimon A, Padan E. Transmembrane domain IV is involved in ion transport activity and pH regulation of the NhaA-Na^+^/H^+^ antiporter of *Escherichia coli*. Biochemistry 41: 609-617 (2002).
2. Olkhova E, Kozachkov L, Padan E, Michel H. Combined computational and biochemical study reveals the importance of electrostatic interactions between the "pH sensor" and the cation binding site of the sodium/proton antiporter NhaA of Escherichia coli. Proteins 76: 548-559 (2009).
3. Alkoby D, Rimon A, Budak M, Patino-Ruiz M, Calinescu O, et al. NhaA Na+/H+ antiporter mutants that hardly react to the membrane potential. PloS one 9: e93200 (2014).
4. Fukada, H. & Takahashi, K. Enthalpy and heat capacity changes for the proton dissociation of various buffer components in 0.1 M potassium chloride. *Proteins* 33, 159-166 (1998).
